# Organizing the Donation of Convalescent Plasma for a Therapeutic Clinical Trial on Ebola Virus Disease: The Experience in Guinea

**DOI:** 10.4269/ajtmh.15-0890

**Published:** 2016-09-07

**Authors:** Alexandre Delamou, Nyankoye Yves Haba, Almudena Mari-Saez, Pierre Gallian, Maya Ronse, Jan Jacobs, Bienvenu Salim Camara, Kadio Jean-Jacques Olivier Kadio, Achille Guemou, Jean Pe Kolie, Maaike De Crop, Patricia Chavarin, Chantal Jacquot, Catherine Lazaygues, Anja De Weggheleire, Lutgarde Lynen, Johan van Griensven, for the Ebola-Tx Consortium

**Affiliations:** ^1^ Centre National de Formation et de Recherche en Santé Rurale de Maferinyah, Forécariah, Guinea; ^2^ Institute of Tropical Medicine, Antwerp, Belgium; ^3^ Centre National de Transfusion Sanguine, Conakry, Guinea; ^4^ Etablissement Français du Sang, La Plaine Stade de France, France; ^5^ Department of Microbiology and Immunology, Katholieke Universiteit Leuven, Leuven, Belgium; ^6^ Association des Personnes Guéries et Affectées d'Ebola en Guinée, Conakry, Guinea; ^7^ Etablissement Français du Sang Auvergne-Loire, France; ^8^ Etablissement Français du Sang Alpes-Méditerranée, France

## Abstract

Although convalescent plasma (CP) transfusion was prioritized among potential Ebola treatments by the World Health Organization, there were concerns on the feasibility of its implementation. We report on the successful organization of donor mobilization and plasma collection as part of the Ebola-Tx clinical trial from November 2014 to July 2015 in Conakry, Guinea. Project implementation registers, tools and reports, mission reports, and minutes of research team meetings were used to reconstruct the sequence of events on how donor mobilization was organized, plasmapheresis was set up, and how effective this approach was in collecting CP. An initial needs assessment of the Guinean National Blood Transfusion Center resulted in targeted training of staff on site, resulting in autonomy and independent production of CP within 3 months. The Conakry Ebola Survivors Association played a direct role in donor mobilization and organization of CP donations. A total of 98 Ebola survivors were screened for plasma donation, of which 84 were found eligible for plasmapheresis. Of these, 26 (30.9%) were excluded. The remaining 58 donors made a total of 90 donations, corresponding to 50.9 L of CP. This sufficed to treat the 99 eligible patients enrolled in the trial. Within a poor resource emergency context, transfusion capacity could be rapidly improved through the strengthening of local capacities and gradual transfer of skills coupled with active involvement of Ebola survivors. However, large-scale plasma collection or multisite studies may require further adaptations of both strategy and logistics. The Ebola-Tx trial was funded by the European Union and others.

## Introduction

The present outbreak of Ebola virus disease (EVD) in west Africa is the largest ever recorded.^1^ In September 2014, the World Health Organization (WHO) prioritized convalescent whole blood (CWB) and convalescent plasma (CP) transfusion among potential Ebola therapies in the hope that they could rapidly be implemented if proven safe and effective.^2^ WHO called for proper testing of these therapies in clinical trials rather than compassionate use,^3^ and developed an interim CWB and CP guideline for patients with EVD as an empirical treatment during outbreaks.^4^

Although WHO did issue some guidance and recommendations on how to organize CWB and CP collection during the EVD outbreak, these were not field tested for EVD. For a number of reasons, doubts and concerns were raised by actors in and outside of Guinea on the feasibility to rapidly organize such a complex intervention in the middle of an overwhelming EVD outbreak. First, national blood transfusion services are poorly developed in the affected countries, and plasma collection and processing is not routinely done. This would thus require the rapid introduction of new technology, which adhered to international standards necessary for a clinical trial, during a medical emergency. Similarly, for reasons primarily linked to culture, voluntary blood donation remains generally uncommon in west Africa. The Guinean Ebola context was characterized by community reluctance and mistrust toward official health education EVD messages, aggravated by rumors and fear about blood donation (e.g., the suspicion that international associations were collecting and exporting blood for financial gain).^5^–^7^ Because of the stigma attached to Ebola survivors, they could be reluctant to volunteer as donors.^7^ In addition, there were concerns that plasma collection procedures were too complex to implement in the context of EVD and potentially risky for donors, patients, and health-care workers.^8^,^9^,^10^

Funded by the European Union, *The emergency evaluation of SP to treat EVD in Guinea* (Ebola-Tx) was one of the consortia that engaged in evaluating CP for treatment of EVD during the 2014–2015 outbreak. This consortium undertook a phase 2/3, open-label, nonrandomized, clinical trial, to evaluate CP versus standardized supportive care in confirmed EVD patients in Conakry, Guinea. The main results of the trial have been reported previously showing that the use of CP to treat EVD patients is safe but was not associated with a significant improvement in survival.^11^

In this article, we report on the successful organization of donor mobilization and plasma collection as part of the Ebola-Tx clinical trial in Donka Hospital, Conakry, Guinea. Our experience and lessons learned could be of value for future studies on CP for EVD and other infectious diseases.

## Settings and Methods

The Ebola-Tx trial, sponsored by the Institute of Tropical Medicine-Antwerp, Belgium, was designed to assess the feasibility, safety, and efficacy of CP treatment of EVD.^11^ Survival in patients receiving CP and supportive care was compared with historical patients treated with supportive care only. We refer to a previous publication for detailed information.^11^ The key partners involved in the plasma collection were the Guinean National Blood Transfusion Center (CNTS) and the Etablissement Français du Sang (EFS).

Laboratory-confirmed EVD patients, using reverse transcription polymerase chain reaction, were consecutively administered two units (200–250 mL each) of ABO-compatible pathogen-reduced CP, originating from two different donors (Intercept Blood System for Plasma, Cerus Europe B.V., Amersfoort, The Netherlands). Donor blood was screened for the human immunodeficiency virus (antigen + antibody), hepatitis C virus (antibody), hepatitis B surface antigen (HBsAg), and syphilis as per national requirements, in addition to pathogen inactivation.

Project implementation registers, tools and reports, mission reports, and minutes of research team meetings were used to reconstruct the sequence of events on how donor mobilization was organized, plasmapheresis capacity was established, and how effective this approach was in collecting CP.

For donor mobilization, the Ebola-Tx approach was compared with recommendations made by the WHO.^12^ The WHO guideline identifies three strategies to engage, recruit, and retain CWB and CP donors. First, donor engagement, which includes reaching out to communities, potential donors, and stakeholders to reduce stigma and facilitate people recovered from EVD to donate CWB/CP. Second, the education and recruitment of people recovered from EVD as potential blood donors for CWB and CP, with the provision of quality donor care and services. Third, the retention of donors on a regular basis and in the long term as general blood donors, if the strategy of transfusing CWB/CP proves to be effective.

The study protocol of the Ebola-Tx trial received formal ethical approvals from the national ethics committee in Guinea, the institutional review board of the Institute of Tropical Medicine, and the ethics committees of the Antwerp University Hospital, the London School of Hygiene and Tropical Medicine, Médecins Sans Frontières, and the WHO.

## Results

### Setting up plasmapheresis capacity.

In November 2014, EFS and the Flemish Red Cross conducted a needs assessment of the CNTS in Conakry. Gaps identified included the lack of robust and continuous electricity supply; limited resources, in particular for hygiene and infection control; the maintenance of the cold chain; and digitalization of data. Insufficient knowledge and practice of biosafety measures, informed consent procedures and good clinical and laboratory practices, and weak documentation practices were also reported. Attention was drawn to the significant proportion of blood donors with a positive serology (especially for hepatitis B virus [HBV]). Positive factors included the motivation of the CNTS team, existence of notions and documents about quality in blood transfusion activities, and fully automated screening for transfusion-transmitted infections (TTIs) with equipment identical to those in use in European blood bank laboratories.

In the same time, the Bill and Melinda Gates Foundation (BMGF) donated a fully equipped bus for plasmapheresis (plasmapheresis bus) to the Government of Guinea, who placed it at the disposition of CNTS. The bus was equipped with four functional plasmapheresis units (PCS^®^2 Plasma Collection System; Haemonetics S.A., Signy Center, Switzerland). Because of electrical incompatibility, the bus was adapted to the Guinean national grid by the joint efforts of Electriciens Sans Frontières and ClinicalRM (a clinical research organization funded by BMGF). All materials in the bus were translated from English to French.

**Figure 1.**
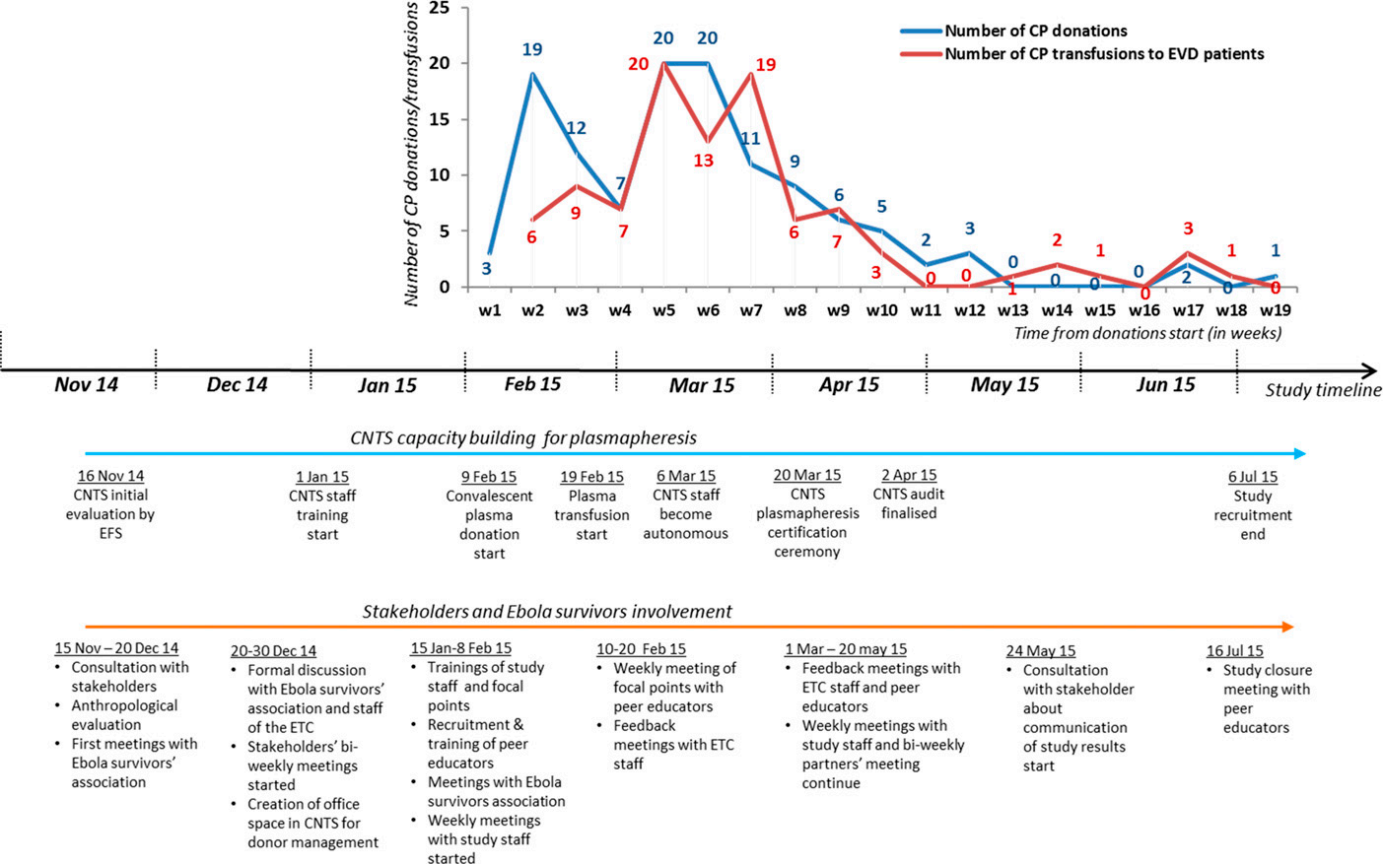
Timeline for stakeholders involvement and plasma production during the Ebola-Tx Trial in 2015, Conakry, Guinea. CNTS = National Blood Transfusion Center (Centre national de transfusion sanguine) CP = convalescent plasma; ETC = Ebola treatment center.

Once the bus was operational, the CNTS staff was trained by EFS teams in screening for TTIs, and the collection, inactivation, preparation, and storage of plasma. Between January 1 and April 2, 2015, EFS teams consisting of two experts rotated weekly in Conakry to train CNTS staff on plasmapheresis, implementation of specific procedures, and documentation (Figure 1

). Although practical training started with donations from non-Ebola survivors, it rapidly switched to Ebola survivors in the second week of February. The composition and roles of CNTS units involved in the Ebola-Tx Trial is detailed in Table 1.

Training allowed CNTS staff to gradually work independently, until they were functioning autonomously. An independent audit mission from April 2, 2015 concluded that “the motivated and disciplined CNTS staffs trained by the EFS team are now autonomous in plasma collection, and the preparation and storage of plasma treated with the Amotosalem pathogen inactivation technique.” Logistic supports provided to CNTS to strengthen CP management include the provision of a powerful generator, cold chain equipment with electronic temperature monitoring, and the standardization of procedures for the transport of CP to the Ebola treatment center (ETC).

### Donor engagement.

The approach for donor engagement among Ebola survivors is detailed in Table 2 and the chronology of donors' involvement and production of qualified CP are presented in Figure 1.

In November 2014, an anthropological pretrial assessment was carried out to support the understanding of the context, the acceptability of the EVD therapies, and the expectations of stakeholders.^13^ Stakeholders included organizations involved in the implementation of the trial, donors, EVD patients and their families, disease surveillance teams, and representatives of the community. The evaluation demonstrated that communication with stakeholders, to understand and follow up what people thought, felt, perceived, and how they acted during the EVD outbreak and consequential health control activities, was and would remain an essential element of ongoing trial activities. The evaluation also emphasized the need to include people affected by EVD in the communication strategy.

A series of consultations with stakeholders were organized, which resulted in the definition of a four-prong strategy for the mobilization and support of CP donors.

First, the Conakry Ebola Survivors Association (APEGUAEG) was identified early on as key to mitigating the mistrust in blood-related activities and stigma of Ebola survivors in local communities, including communication about the study and the mobilization of potential donors. The APEGUAEG supported the identification among their members of peer educators—for community work and donor recruitment—and persons with medical backgrounds as contact persons, who would coordinate the work of peer educators with the Ebola-Tx team. Second, a training program, including a visit to the plasmapheresis bus, targeted peer educators, and different teams working in the ETC such as the health promotion, water and sanitation, and the psychosocial teams, to help demystify the project. Third, communication tools, such as “question and answer” sheets, were developed to ensure effective and streamlined communication and to counter rumors and misinformation. Fourth, a communication feed-back loop was established through regular meetings with the peer educators, closely linked to the community and Ebola survivors. In addition, frequent interaction with all relevant stakeholders was ensured, including regular communication with the ETC, which allowed for the fine-tuning of the trial communication and community support strategy and reactivity toward newly identified needs or rumors.

### Yield of qualified CP.

**Figure 2.**
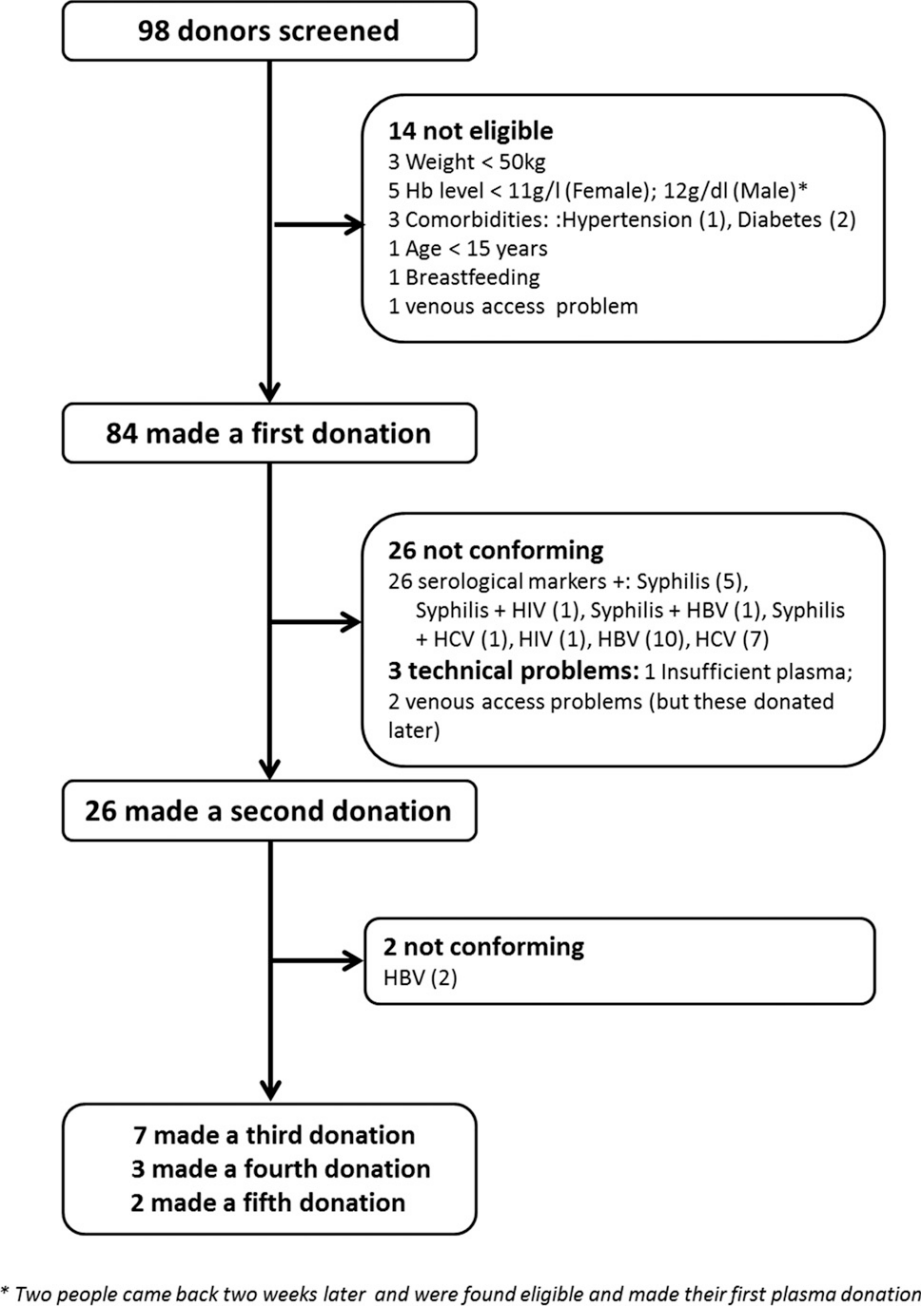
Flow chart summarizing the yield of plasma donation in the Ebola-Tx Trial, in 2015 in Conakry, Guinea. F = female; HBP = high blood pressure; HBV = hepatitis B virus; HCV = hepatitis C virus; HIV = human immunodeficiency virus; M = male.

Plasma donation from Ebola survivors started on February 9, and finished on July 7, 2015, at CNTS in Conakry. Figure 2

provides an overview of plasma donation throughout the study. A total of 98 Ebola survivors were screened for plasma donation of which 84 (85.7%) were eligible and made a first donation. The most frequent reasons for ineligibility were low hemoglobin level (five out of 14), diabetes (two out of 14), hypertensive disorder (one), and body weight less than 50 kg (one). Of the 84 donors who made a first donation, 26 (30.9%) did not conform, meaning their plasma was not suitable for use in the study. Reasons for nonconforming included the presence of serological markers of TTIs (*N* = 26, 30.9%). For three additional donors, the first donation was not successful due to technical problems. Donors tested positive for TTIs were managed according to national guidelines (they were invited individually to know their TTI screening results and were offered further diagnostic testing and care at CNTS or another hospital). Of the 58 donors with conforming CP, 26 (44.8%) made a second donation, two of which did not conform (HBsAg positive). Seven donors made a third donation, three made a fourth donation, and two donors made a fifth donation. Up to 600 mL were collected per donation, depending on the weight of the donor. A minimum of 2-week interval was observed between every donation. Each conforming plasma was divided in portions of 200–300 mL.

The 98 donors made a total of 135 visits, resulting in 121 plasma donations, of which 90 were qualified for transfusion (74.4%). The total volume of conforming CP collected was 50.9 L.

As can be seen in Figure 1, the rate of CP collection followed the patient recruitment rate. For all of the 99 patients treated with CP in the trial, ABO-compatible CP was available. CP donations were planned in function of the amount of CP in stock for each blood group. The CP stock management was done at CNTS, via a continuously updated CP list which was shared in real time with the ETC. Donor identity was kept confidential and only donation identifier, ABO group, volume, and collection dates were provided to the ETC. When necessary, specific donor types were actively solicited to prevent anticipated stock ruptures, particularly if several patients with an identical blood group were EVD confirmed at the same time. All donors were offered a lunch and their transportation costs were reimbursed.

## Discussion

Ebola-Tx is the first study to document the organization of CP donation for an EVD therapeutic clinical trial, at large scale, in an emergency context. Despite of difficulties and delays resulting from the poor resource setting and emergency nature of the trial, it was possible to strengthen local capacities and transfer skills resulting in successful plasmapheresis in CNTS.^14^–^16^

Factors that facilitated this achievement were 1) the availability of international guidelines such as those provided by the WHO,^12^ 2) the motivation of local staff, 3) donation of the plasmapheresis bus, 4) support of EFS in local capacity building, and 5) timely and rapid assessment, which accurately identified the main needs and challenges for capacity building at the CNTS, followed by a jointly established timeline.

The adopted approach resulted in CNTS staff mastering the plasmapheresis process in 3 months. The independent audit emphasized that a complete technique had been implemented pragmatically and can serve as an inspiring example of a successful local capacity-building approach in the middle of an emergency.

Donor recruitment among people recovered from Ebola followed WHO recommendations.^12^ The 90 conforming plasma donations were used to treat 99 EVD-confirmed patients, the highest EVD trial recruitment ever achieved.^17^ In addition, the study provided CP for all EVD-confirmed eligible patients presenting at the ETC in Donka Hospital. In this trial, 28 out of 98 (28.6%) screened donations were excluded for TTIs, in line with the high prevalence of these infections in the local context.^6^,^18^ Concerning the two donors tested positive for HBV on the second donation, their first donations were reevaluated and found negative suggesting incident HBV infections. Their samples were sent abroad for further analysis and the case is followed up by the CNTS.

Key strategies to involve and retain donors included 1) the initial anthropological assessment that improved our understanding of the stakeholders expectations and concerns; 2) involvement and close collaboration with the APEGUAEG Ebola survivor association in the recruitment and supervision of focal points and peer educators among Ebola survivors; 3) a global but low profile communication strategy operating in Conakry and neighboring cities targeting survivors, implementing partners, families of EVD patients, disease surveillance and social mobilization teams; 4) strong leadership of the trial coordination team that built trust with survivors. Although the available WHO recommendations supported general planning and strategy, the experience throughout the trial demonstrated the importance of integrating local context and cultural considerations to guide strategic choices to implement a study in an emergency setting, such as the Ebola-Tx trial. For instance, although WHO recommend predonation screening for TTI, in our context, Ebola survivors insisted they should be given the possibility of having TTI screening and plasma collection done at the same time.

However, applying the WHO selection criteria,^4^ the number of donors screened during the trial was lower than expected. In data available for this trial up to March 31, 2015, donors only represented 7% (98/1,418) of nationwide survivors and 18% (98/547) of those in the Conakry area (donors came from Conakry, Coyah, Forecariah, Dubreka, and Kindia).^19^ In addition, only 46.4% (26/55 donors) of donors with conforming CP made a second donation on request. Although there was sufficient CP to treat all trial patients, this demonstrates that limiting factors to recruit and retain donors existed, and sudden increase in EVD case load could have led to stockouts. More efficient approaches would be needed for large-scale trials or national scaling up of the intervention.

A study by Wong and others in 2009, for the use of CP to treat influenza A (H1N1) infection,^20^ identified a number of practical difficulties in plasma collection. The Ebola-Tx study identified similar challenges including 1) contacting and reaching people by phone, 2) refusal to participate in a voluntary donation, 3) limiting inclusion criteria, and 4) time constraints.^21^ Time constraints included a mismatch in donor availability and CNTS working hours. Because of work constraints, travel, and stigma, certain donors preferred donating plasma early morning, late evenings, or at weekends, times when CNTS staffs were not available. Many survivors gave incorrect addresses on discharge from the ETC making tracing difficult. Reasons for this are thought to include stigma, fears of discrimination, and social reluctances and rumors about blood trafficking by foreign bodies.^5^,^6^ In addition, although Ebola-Tx complied with Guinea law, which forbids remuneration for blood/plasma donations, other research groups present in Guinea at the time of the trial did offer money to EVD survivors for blood sample collection. These factors are assumed to have contributed to the reduced rate of plasma collection.

## Conclusions

Despite the challenges encountered, the Ebola-Tx trial was able to collect, qualify, and provide the Donka Hospital ETC with sufficient CP to treat all EVD-confirmed patients until the end of the trial. CP donation was successfully organized at the Conakry CNTS in Guinea as part of the implementation of the Ebola-Tx trial. Regulations such as non-remunerated donations were respected within a highly pressured context with uncontrollable external factors. Despite the poor resource and emergency setting, local capacity for high-standard technology such as plasmapheresis with a pathogen inactivation technique was feasible when adequately planned in the early design phase. Identifying and involving stakeholders with clearly defined responsibilities along with a transparent system of communication and feedback and a strong local leadership were key factors to success. However, large-scale plasma collection or multisite studies may require further adaptations of both strategy and logistics.

## Figures and Tables

**Table 1:** Description and roles of CNTS units involved in the Ebola-Tx Trial in 2015, Conakry, Guinea

CNTS units	Description	Roles and responsibilities
Plasma collection unit	Composed of four staff from the CNTS blood collection unit	• Welcome donors with focal points
		• Conduct predonation counseling and interviews
		• Medical visit (blood pressure, weight, hemoglobin)
		• Informed consent
		• Conduct and supervise plasma donation and put the collected plasma in the fridge inside the bus
		• Collect blood samples at the beginning of the donation and transfer the sample to the appropriate units (hematology and serology) and document it
		• Check battery and fuel level and make requests to the administration if needed
		• Ensure cleanliness of the bus
		• Maintain unit logs and registers up to date
Hematology unit	Composed of two staff from the CNTS hematology unit and one laboratory officer recruited and positioned at the Hemorrhagic Fever Laboratory (blood typing for Ebola patients)	• Receive blood samples from the plasma mobile bus
		• Perform blood typing for donors, fill the register, and communicate the results to the preparation unit
		• On request (in case of positive PCR in suspected Ebola cases received at the ETC), go to the Hemorrhagic Fever Laboratory and perform the blood typing for those positive/confirmed EVD cases following safety procedures
		• Communicate the results to the study staff in the ETC and the study field coordination.
Serology unit	Composed of two staff from the CNTS serology unit	• Receive blood samples from the plasma mobile bus (from donors)
		• Perform serological tests for HIV, HBV, HCV, and syphilis using the Architect machine for donors, fill the register, and communicate the results to the preparation and distribution unit (conform or not conform for transfusion)
		• Check and put the samples to be sent in France (for further analyses that are not available in Conakry) in the study's sample freezer
Preparation and distribution unit	Composed of two staff from the CNTS preparation and distribution unit	• Receive/collect results of blood typing and serology from the corresponding units
		• Report the results in the plasmapheresis register
		• Conduct the inactivation process using Amotosalem pathogen inactivation technique with the collected plasma inside the bus and carry the inactivated plasma out of the bus and put it in the fridge for nontested plasma
		• If the donor is conform, divide the plasma into small bags, label, and transfer the plasma to the conforming plasma fridge
		• Store the plasma between 2°C and 8°C for 40 days in the conforming plasma fridge after which frozen at −30°C
		• If the donor is not conform, transfer the plasma to the nonconforming plasma freezer
Quality control unit	Composed of the CNTS quality control officer	• Conduct regular quality checks on study registers and logs
		• Control the temperature for all study related fridges and freezer and fill the temperature log
		• Inform the staff and the field coordination of any problem encountered or discovered and follow up with the implementation of any corrective measure taken

CNTS = National Blood Transfusion Center (Centre national de transfusion sanguine); ETC = Ebola treatment center; EVD = Ebola virus disease; PCR = polymerase chain reaction.

**Table 2:** WHO steps of involving Ebola survivors in plasma donation and its adaptation to the context of the Ebola-Tx Trial in 2015 in Conakry, Guinea

WHO steps	Ebola-Tx: what was done?	Why/how?	Who was involved?
1. Community and donors' engagement and support (WHO Strategy 1)	• Anthropological evaluation	• To understand the context of the study, expectations from stakeholders, challenges and suggestions to make the study acceptable	ITM, CNTS, Maferinyah Training and Research Center, Laboratoire des Fièvres Hémorragiques de Guinee, Ministry of Health, National Ebola Task Force, MSF, international institutions such as WHO local office, Ebola survivors–APEGUAEG, families of Ebola patients, and the staff of the ETC.
	• Consultations with stakeholders		
	• Reaching and engaging communities	• Conakry disease surveillance teams	Conakry Regional Health Directorate
		• MSF health promotion teams	Study field coordination
			Anthropological team
			MSF
	• Identification of the survivors' association in Conakry (APEGUAEG)	• To identify the needs and provide support to the association	Study field coordination, APEGUAEG
	• Discussions about ways to get the association involved in the study	• To define common strategies to inform and recruit donors	Anthropological team
	• Identification of focal points and peer educators together with the association	• To promote effective participation of Ebola survivors in the study, develop common key messages, and refine the study communication strategy	Study field coordination
			APEGUAEG
			CNTS
			Anthropological team
2. Education and recruitment of plasma donors (WHO Strategy 2)	• Theoretical and practical training of focal points and peer educators	• To reinforce focal points' and peer educators' skills, promote plasma donation, reduce donors' stigmatization, and promote ownership of the project	Study field coordination, APEGUAEG
			MSF, anthropological team
			CNTS
3. Retention of plasma donors (WHO Strategy 3)	• Encouraging donors to come back	• Friendly welcoming, transparency, and privacy keeping	Focal points, peer educators, study field coordination, anthropological team
		• Close follow-up and regular update on the study	
		• Frequent (weekly) meetings with peer educators and focal points	

APEGUAEG = Conakry Ebola Survivors Association (Association des Personnes Gueries et Affectées d'Ebola en Guinée); CNTS = National Blood Transfusion Center (Centre National de Transfusion Sanguine); ETC = Ebola treatment center; EVD = Ebola virus disease; ITM = Institute of Tropical Medicine; MSF = Medecins Sans Frontieres; PCR = polymerase chain reaction; WHO = World Health Organization.
